# A novel homozygous nonsense *ZP1* variant causes human female infertility associated with empty follicle syndrome (EFS)

**DOI:** 10.1002/mgg3.1269

**Published:** 2020-04-23

**Authors:** Qianhua Xu, Xiaoli Zhu, Madiha Maqsood, Wenqing Li, Xianhong Tong, Shuai Kong, Fengsong Wang, Xiaoman Liu, Zhaolian Wei, Zhiguo Zhang, Fuxi Zhu, Yunxia Cao, Jianqiang Bao

**Affiliations:** ^1^ Reproductive Medicine Center Department of Obstetrics and Gynecology the First Affiliated Hospital of Anhui Medical University Hefei China; ^2^ NHC Key Laboratory of Study on Abnormal Gametes and Reproductive Tract Hefei China; ^3^ Anhui Province Key Laboratory of Reproductive Health and Genetics Anhui Medical University Hefei China; ^4^ Division of Life Sciences and Medicine The First Affiliated Hospital of USTC University of Science and Technology of China Anhui China; ^5^ School of Life Science Anhui Medical University Hefei China

**Keywords:** empty follicle syndrome (EFS), in vitro fertilization (IVF), whole‐exome sequencing (WES), zona pellucida (ZP), *ZP1*

## Abstract

**Background:**

Empty follicle syndrome (EFS) is a rare but severe condition in which no oocyte is recovered in female patients undergoing in vitro fertilization (IVF) after sufficient ovarian response to hormonal trigger. Accumulating evidence highlights the genetic basis of EFS occurrence.

**Methods:**

In this study, we report a patient with primary infertility showing the characteristics of EFS from a consanguineous family. Under the treatment of assisted reproductive technique (ART), no oocyte was retrieved following the aspiration of mature follicles. Through whole‐exome sequencing (WES), we discovered a novel recessively transmitted mutation in *ZP1* (c.769 C>T, p. Q257*).

**Results:**

In vitro Co‐immunoprecipitation assays showed that mutant ZP1 protein failed to interact with either ZP2 or ZP3, which explains the degenerated oocytes in the patient with EFS.

**Conclusion:**

Together, our data further expand the spectrum of *ZP1* mutations that are associated with human EFS and thus provide novel insight into the diagnosis of EFS patients.

## Graphical abstract

ZP1 mutation causes empty follicle syndrome.

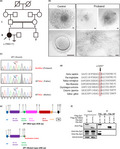

## 1 INTRODUCTION

Retrieval of high‐quality oocytes is an essential step for in vitro fertilization (IVF), which is usually achieved through ovarian stimulation via vaginal puncture. During this process, superovulated oocytes, which are centrally located in the cumulus–oocyte complexes (COCs), can be manually isolated from follicular fluid. In rare occasions, as documented by Coulam three decades ago (Coulam, Bustillo, & Schulman, 1986), no oocyte can be recovered from the COCs following adequate ovarian response to stimulation and follicular development, which is referred to as empty follicle syndrome (EFS; Revelli et al., 2017). In the clinics, EFS is in general categorized into two classes: ‘false’ EFS (FEFS), which are linked to low circulating levels of β‐human chorionic gonadotropin (β‐hCG) mainly due to inappropriate timing or dosage of hCG administration, and ‘genuine’ EFS (GEFS), of which the etiology remains obscure as yet (Mesen et al., 2011; Revelli et al., 2017; Zreik et al., 2000).

Over the past years, it has been estimated that the incidence of GEFS occurs around 0.016% among patients undergoing IVF (Mesen et al., 2011; Revelli et al., 2017; Zreik et al., 2000). While some previous studies have suggested that dysfunctional folliculogenesis and ovarian ageing are involved in GEFS, more recent efforts implicated substantial genetic factors contributing to GEFS. For examples, the sporadic inversion of chromosome 2 and mutations in luteinizing hormone/choriogonadotropin receptor (*LHCGR* [MIM: 152790]) were found as the causative factors in patients with GEFS (Chen et al., 2018; Yuan et al., 2017). A single recurrent heterozygous mutation (*ZP3* [MIM: 182889] c.400 G>A) was identified to be responsible for GEFS in three unrelated families (Chen et al., 2017). Notably, two groups independently discovered compound heterozygous and homozygous mutations in *ZP1* (MIM: 19500) gene in GEFS patients from unrelated families (Dai, Chen, et al., 2019; Sun et al., 2019). During folliculogenesis, an oocyte is developing within a unique layer of extracellular matrix coat, called zona pellucida (ZP). Interestingly, detailed examination of ovary biopsy revealed normal preantral folliculogenesis but with aberrant ZP assembly in GEFS patients (Dai, Chen, et al., 2019). These findings further corroborated the existence of GEFS, and thus prompted genetic disorders of ZP as causative factors of GEFS.

Human genome encodes four sulfated ZP glycoproteins, namely, ZP1–4, while mouse ZP family comprises only three members: ZP1–ZP3 (Wassarman & Litscher, 2018). It is generally believed that ZP2 and ZP3 are predominant building blocks that form the ZP filaments, whereas ZP1 functions by covalently crosslinking ZP filaments into a three‐dimensional matrix. As a whole, well‐organized ZP filaments make up a firm extracellular coat that not only safeguards the oocyte development but also accommodates appropriate timing of oocyte–sperm recognition, for example, avoiding the polyspermy (Wassarman & Litscher, 2018). The functional consequence of ZP4 has not been appreciated as yet in humans.

In this study, an autosomal homozygous mutation in *ZP1* (MIM: 195000) gene was identified in a consanguineous family by whole‐exome sequencing (WES). This mutation was recessively transmitted from the father and the mother who were first cousins. We speculated that this homozygous nonsense mutation led to the primary infertility as a result of the failure of ZP assembly and degeneration of oocytes.

**FIGURE 1:**
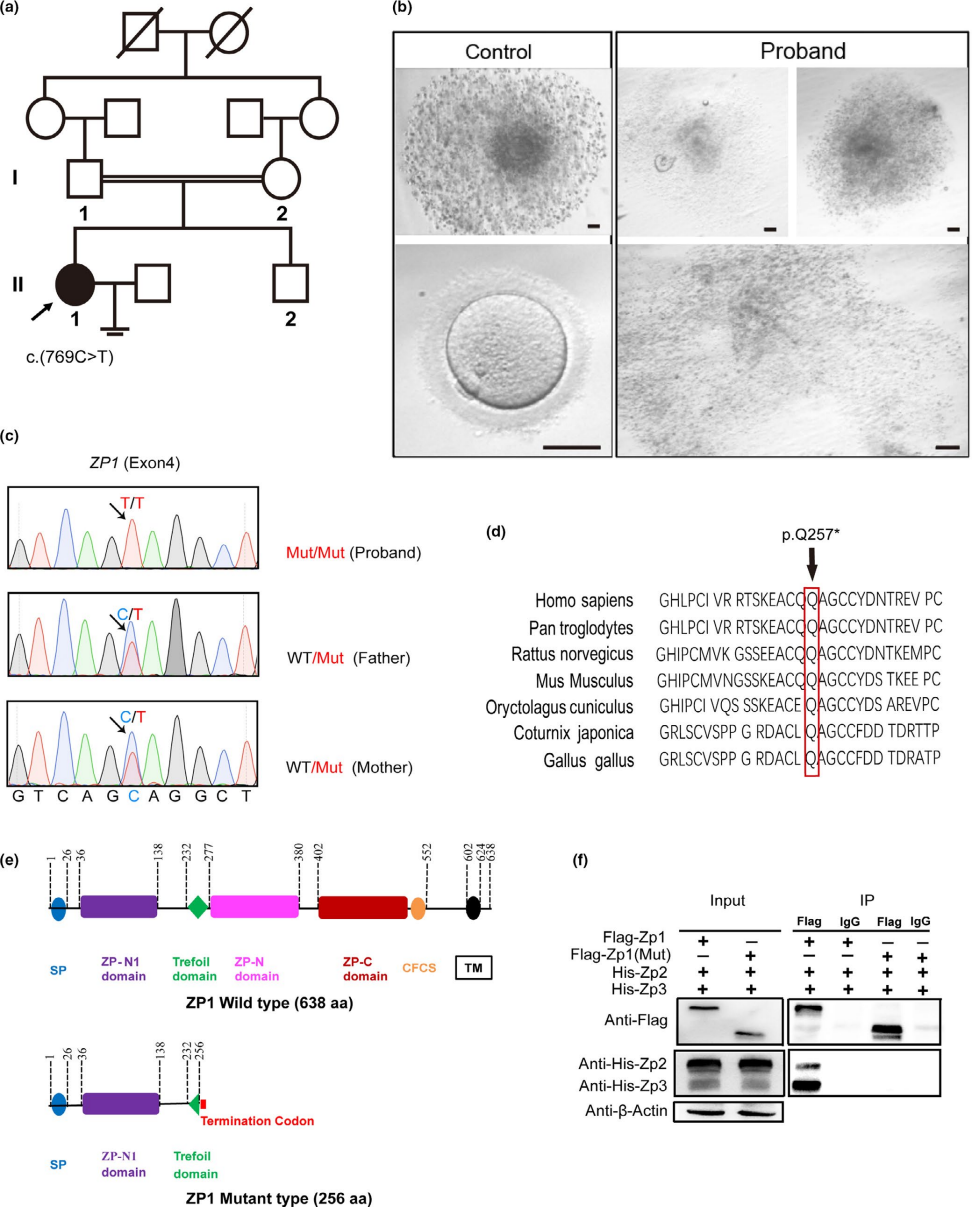
The homozygous nonsense ZP1 mutation (c.769 C>T, p. Q257*) in a woman with empty follicle syndrome from a consanguineous family. (a) The pedigree information of the consanguineous family. The arrow denotes the proband (II‐1). Her father and mother are first cousins, and carried a single (c.769 C>T) mutant allele, respectively. (b) The characteristic morphology of COCs and oocytes aspirated from the follicular fluid from the proband and control as indicated, following a long GnRH agonist treatment protocol. No clear oocytes, but only ooplasm‐like fragments, were identified in the proband. In contrast, the control COCs exhibited well‐expanded granulosa cells surrounding the central oocyte. After removal of the granulosa cells by hyaluronidase, a mature oocyte at MII with a thick layer of ZP displays clear, homogeneous granularity in the cytoplasm. Bar = 60 μm. (c) Validation of the ZP1 mutation (c.769 C>T) by Sanger Sequencing on the blood DNA samples from the proband and her parents. PCR primers were designed against exon 4 of human ZP1 (Table 2). (d) Multiple sequence alignment showing amino acid conservation of ZP1 orthologs across different species. Amino acid highlighted by red box points to glutamine that was substituted by a premature STOP codon in ZP1 (p. Q257*) observed in the proband. (e) Schematic diagram of the human ZP1 protein structure. Full‐length human ZP1 (638 aa) comprises of N‐terminal signal peptide (SP), ZP‐N1 domain, Trefoil domain, ZP‐N domain, ZP‐C domain, CFCS, as well as a C‐terminal transmembrane (TM) domain. By comparison, ZP1 mutation (c.769 C>T) in the proband created a C‐terminal truncated ZP1 protein (256 aa) due to occurrence of a premature STOP codon at aa 257, missing the domains starting from ZP‐N. (f) Co‐immunoprecipitation (Co‐IP) assay. Mouse full‐length Zp1–3 and corresponding mouse truncated Zp1 plasmids were co‐transfected into 293T cells, and were co‐expressed ectopically as tagged protein as indicated, followed by Co‐IP assay. 10% cell lysates were run on SDS‐PAGE gel as input control. Isogenic mouse IgG served as a negative control. While full‐length Zp1 proteins reproducibly pulldown both WT Zp2 and Zp3 proteins, mutant truncated Zp1 observed in the proband was not readily detected to interact with either Zp2 or Zp3

The proband (family member II‐1) from this consanguineous family was a 28‐year‐old woman, with a diagnosis of unexplained primary infertility after 2 years of cohabitation with her husband (Figure 1a) recruited from the First Affiliated hospital of Anhui Medical University. She had a younger brother who was not married. This patient also had regular menstrual cycle (26–28 days on average per month) since menarche at the age of 16. The semen analyses of her husband revealed normal values of a variety of parameters according to WHO's standards (Data not shown). In addition, this couple had no history of significant familial illness or aberrant karyotypes by chromosome examinations. Her basal levels of sex hormones fell within normal range and other infertility‐related evaluations did not unveil any anomalies (Table 1). Given this unexplained primary infertility, she received one cycle of IVF treatment, with a long gonadotropin‐releasing hormone (GnRH) agonist protocol. Following the identification of 6 leading follicles (≧18 mm) among a total of 12 follicles, along with a serum level of estradiol at 17,622 pmol/L, a single dose of human chorionic gonadotropin (hCG; 250 µg) was administered. Oocyte retrieval was carried out 36 hr after hCG trigger. Seven COCs were isolated by a pasteur pipette, but no single recognizable oocyte was identified. Only partial degenerated oocyte cytoplasts were identified with the help of a pasteur pipette (Figure 1b). The β‐hCG level on the day of oocyte retrieval was 76.3 IU/L, which strongly prompted an GEFS phenotype. In contrast, the control COCs collected from a fertility‐proven donor possessed radiating and well‐expanded cumulus cells. The female donor was from an infertile couple with female tubal factor, the couple presented with secondary infertility over 3 years after they had the first child. The hormone levels of the female donor were normal and the semen analysis of her male partner showed no abnormality. Tubal surgery was not a realistic option for them, IVF treatment was chosen. Upon removal of the cumulus cells, the control oocyte exhibited a clear cytoplasm with homogeneous granularity, surrounded by a thick layer of zona pellucida (Figure 1b).

**TABLE 1:** The parameters during ART cycle

Protocols	GnRH agonist (long protocol) cycle 1
Duration of stimulation (d)	10
Total gonadotropin dose (IU)	1,500
Basal hormones (normal reference range)	
hFSH (IU/L)	6.97 (2.5–10.2)
hLH (IU/L)	5.31 (1.9–12.5)
E2 (pmol/L)	446 (231–606)
Prog (nmol/L)	1.9 (0.48–4.45)
PRL (ng/ml)	27.3 (2.8–29.2)
Testo (nmol/L)	2.15 (0.48–2.64)
Hormones assay on day of HCG administration	
Serum LH level (IU/L)	1.02
Serum E2 level (pmol/L)	17,622
Serum progesterone level (nmol/L)	3.03
No. of leading follicles (18 mm)	6
No. of total follicles	12
β‐hCG level on the day of oocyte retrieval (IU/L)	76.3
Ovulation trigger	
Type of trigger	Single trigger
Drug and dose (µg)	r‐HCG 250

To probe the causative mutations, we performed WES using the DNA extracted from the periphery blood sample in the patient. Agilent Human SureSelect All Exon V6 kit was exploited for exome library preparation, and sequencing was performed on an Illumina NovaSeq 6000 platform. Clean sequencing reads were aligned to the human genome (hg19). Sequence variants, including single‐nucleotide variants (SNVs) and small insertions/deletions (INDELs), were annotated by ANNOVAR pipeline (Wang, Li, & Hakonarson, 2010). Because this patient came from a consanguineous family, we would thus anticipate that a homozygous mutation transmitted from the parents was responsible for the EFS phenotype observed in this proband. Thus, a candidate homozygous variant was selected if it satisfied the criteria as below: (a) it has not been reported, or is a rare variant with a minor allele frequency (MAF) below 1% in four public databases: 1000 genome, dbSNP, gnomAD, and Exome Aggregation Consortium (ExAC); (b) nonsynonymous exonic or splice site variant, or frameshift INDEL, (c) known RNA expression in our in‐house oocyte expression database, and (d) heterozygous variant that was also carried by the parents. This filtering strategy revealed a stop‐gain homozygous variant (c.769 C>T, p. Q257*) in *ZP1* (NM_207341) gene located in 11q12.2, as a highly likely potential pathogenic variant (Table 2). This variant (rs769509601) has not been recorded in 1000 genome database, but with a frequency at 0.00000407 in GnomAD database. Furthermore, Sanger sequencing using blood DNA samples validated that her father and mother individually carried a same single mutant allele (c.769 C>T) as found in the proband (Figure 1c). This mutation rendered a premature stop codon in exon 4 at 769 nucleotide, resulting in a C‐terminally truncated ZP1 protein with 256 amino acids in total, instead of full‐length 638 amino acids. The amino acid wherein glutamine (Q) is mutated resulting in a premature STOP codon, is also conserved across a myriad of organisms (Figure 1d). Mutant ZP1 protein possessed N terminal signal peptide (SP), ZP‐N1 domain, as well as a half of Trefoil domain, missing the ZP‐N, ZP‐C, and the transmembrane (TM) domains as compared with WT ZP1 protein (Figure 1e).

**TABLE 2:** Number of variants filtered through pipeline of whole‐exome sequence analysis

Variants	SNP	INDEL
Coding homozygous variants	8,528	571
Not reported or frequency <1%	31	18
Coding (nonsynonymous exonic or splice site variant)	18	3
SIFT and polyphen prediction	3	1
Known RNA expression in our in‐house oocyte expression database	1 (*ZP1*)	0

To determine the detrimental effect due to *ZP1* (p. Q257*) mutation that are associated with the absence or degeneration of oocytes leading to the GEFS in the proband, we constructed the full‐length WT expression plasmids for mouse Zp1–3 as well as the mutant plasmid containing corresponding mutation in mouse Zp1 as observed in patient. Flag‐tagged WT or mutant Zp1 constructs were simultaneously cotransfected into 293T cells together with His‐tagged Zp2 and Zp3. In vitro Co‐immunoprecipitation (Co‐IP) experiment demonstrated that WT Zp1 was readily able to pull down both Zp2 and Zp3 proteins, whereas truncated Zp1 failed to pull down either Zp2 or Zp3 (Figure 1f), suggesting *ZP1* (p. Q257*) mutation abolished the interaction between ZP1 and ZP2/ZP3. These evidence are in accordance with recent studies, in which truncated ZP1 protein, either ZP1(p.G57Dfs*9) or ZP1(p.I390Tfs*16), both lacking C‐terminal transmembrane (TM) domain, was not able to interact with ZP2 and ZP3 (Sun et al., 2019; Zhou et al., 2019).

In mammals, eggs are universally surrounded by a gel‐like extracellular matrix, called zona pellucida, which plays a significant role in oogenesis, fertilization, and preimplantational embryonic development (Wassarman & Litscher, 2018). During human ZP assembly, ZP2 (MIM: 182888) and ZP3 (MIM: 182889) are abundantly present roughly in equimolar amounts, while ZP1 is the least amount but forms a dimer of identical polypeptide chains linked through intermolecular disulfides. The amino acid sequences of ZP2 and ZP3 are more conserved between 65%–98% across mammal species, whereas ZP1 exhibits a lesser degree of conservation around 40% (Wassarman & Litscher, 2018). The human ZP protein family has a fourth member termed ZP4 (MIM: 613514), whose functions remain enigmatic so far. ZP4 is absent in mouse species in that it is encoded by a pseudo‐gene which is inactive during oocyte development. However, ZP1 is substituted completely by ZP4 in some mammals, for instances, pig, cat, dog, etc. (Wassarman & Litscher, 2018). Previous studies have shown that ZP2 and ZP3 largely serve as building blocks of ZP, while ZP1 covalently crosslinks ZP2/3 filaments, forming a robust extracellular matrix. In agreement with these evidence, *Zp2‐* or *Zp3*‐deficient mice achieved via gene targeting do not possess ZP in the ovulated eggs, and are thus completely infertile (Liu et al., 1996; Rankin et al., 2001). By comparison, *Zp1*‐null mice produced oocytes with thinner, loosely organized ZP, but were still fertile albeit with markedly reduced fecundity (Rankin, Talbot, Lee, & Dean, 1999).

With the assistance of next‐generation exome sequencing, recently, more novel mutations in *ZP1–3* responsible for female infertility were identified in human patients. Lin et al in 2018 reported a homozygous frameshift *ZP2* (MIM: 182888) variant (p.C566Wfs*5), which truncated ZP2 C‐terminal TM domain resulting in thinner ZP, in an infertile woman (Dai, Chen, et al., 2019; Dai, Hu, et al., 2019). A single recurrent missense *ZP3* mutation (c.400 G>A) that is paternally inherited in a large family led to female infertility characterized by recurrent EFS, likely through a dominant‐negative mechanism (Chen et al., 2017). More recently, during the preparation of this manuscript, three independent groups identified one compound heterozygous *ZP1* mutation (p.G57Dfs*9 & p.I390Tfs*16), three homozygous *ZP1* variants (p.Val570Met, p.Arg410Trp, p.His170Ilefs*52), and 6 novel *ZP1* mutations that are tightly associated with empty follicle syndrome and female infertility (Dai, Chen, et al., 2019; Sun et al., 2019; Zhou et al., 2019). In some cases, intact three‐dimensional oocytes without ZP can be observed in *ZP* mutation‐carrying patients, who cannot be classified into GEFS (Zhou et al., 2019). The existence of intact, ZP‐free, but not degenerated, oocytes in those patients is highly likely dependent upon the residual function of mutant ZP proteins, or the complementary role of one copy of the intact allele in heterozygous patients. Indeed, this postulation is in accordance with a recent study, in which Gao et al. demonstrated the definitive dose‐dependent effects of ZP2 and ZP3 in humans and validated in mice (Liu et al., 2017). Therefore, the functional consequence, either ZP‐free oocytes or GEFS, elicited by *ZP* gene mutations, varies among different patients. It is likely that, in general, the more loss of ZP protein sequences (domains), the resultant symptom is more severe, that is, GEFS. Our study, together with recent findings, suggests that there is an imperious demand to explore more potential *ZP* mutations in patients suffering from GEFS.

In summary, we unveiled a novel homozygous *ZP1* variant in a woman with GEFS from a consanguineous family and provided genetic evidence linked to GEFS, thus further reinforcing the existence of empty follicle syndrome. This study expanded the *ZP1* mutation spectrum, and provided a novel glimpse into the heterogeneity of GEFS etiology. Altogether, it suggests that *ZP* mutations, in particular *ZP1*, appear to be “hotspot” as causative factors underlying patients suffering from GEFS. Therefore, identification and characterization of more novel mutations of *ZP* genes in humans would undoubtedly provide more approaches for diagnosis and prenatal genetic screen (PGS), in the clinics in the future.

## 2 MATERIALS AND METHODS

### 2.1 Ethical compliance

This study was reviewed and approved by the ethics board committee at the First Affiliated Hospital of Anhui Medical University as well as the First affiliated hospital of USTC (University of Science and Technology of China).

### 2.2 Study subjects

The proband (female patient) and other family members were recruited from Reproductive Medicine Center of the first affiliated hospital of Anhui Medical University with patients consent. This study was approved by the ethics committee of Anhui Medical University, and is in accordance with the regulation of the first affiliated hospital of University of Science and Technology of China (USTC).

### 2.3 Sanger sequencing

Primers against exon 4 of *Zp1* gene were designed and used for PCR amplification. PCR products were examined by agarose gel electrophoresis and column purified (Sangon Inc), followed by bidirectional sequencing using an ABI 3100 DNA analyzer (Applied Biosystem).

### 2.4 DNA extraction

Peripheral blood samples from human patients were drawn with the informed consent, and preserved in anticoagulant tubes containing EDTA stored in −80°C freezer. Genomic DNA was purified from frozen blood samples using the QIAamp DNA blood Mini kit following the manufacturer's protocol (Qiagen). DNA concentrations and quantities were assessed by a N50 spectrophotometer (Implen).

### 2.5 Plasmids construction

Total RNA was extracted from 10 ovaries from female mice between 4–6 weeks old using Trizol following manufacturer's protocol. First‐strand cDNA was synthesized using ProtoScript II cDNA first strand kit (NEB) with 1 µg of total RNA. The full‐length coding sequence (CDS) for mouse wild‐type *Zp1* (NM_009580.2), *Zp2* (NM_011775.7)*, Zp3* (NM_011776.1), and mutant Zp1 (Zp1Mut,M1‐Q248) was amplified by high‐fidelity PCR enzyme (NEB) using mouse ovary cDNA as templates. PCR products were gel purified and in‐frame cloned into p3xFLAG‐myc‐CMV^TM^‐24 vector through double enzyme digestion (Not I & Bgl II) for wild‐type Zp1 and mZp1Mut (M1‐Q248). CDS for mouse Zp2 and Zp3 was in‐frame cloned into a modified pcDNA3.1 vector with myc‐6His tag and 6His tag, respectively, via homologous recombination. Primers were listed in Table 3. All final constructs were eventually examined by Sanger sequencing to ensure mutation free.

**TABLE 3:** PCR primer sequences

Primer name	Sequence
For cloning	
Zp1 forward primer	gagagcggccgcgATGGCCTGGGGTTGTTTTGTGG
Zp1 reverse primer	gagaagatctctaATATCTGATGCCTTCCCAGAGC
Zp1mut forward primer	gagagcggccgcgATGGCCTGGGGTTGTTTTGTGG
Zp1mut reverse primer	gagaagatctCTACTGACAGGTTTCCTTGGAAC
Zp2 forward primer	ttaagcttggtaccgagctcATGGCGAGGTGGCAGAGG
Zp2 reverse primer	atgagtttttgttcagaaccGTGATTGAACCTTATAGTTCTTTTCTTATACA
Vector PCR for Zp2 F	GGTTCTGAACAAAAACTCATCTCAGA
Vector PCR for Zp2 R	GAGCTCGGTACCAAGCTTAACTAGC
Zp3 forward primer	ttaagcttggtaccgagctcATGGCGTCAAGCTATTTCCTCT
Zp3 reverse primer	tgatggtgatgatgaccaccTTGCGGAAGGGATACAAGGTAG
Vector PCR for Zp3 F	GGTGGTCATCATCACCATCACC
Vector PCR for Zp3 R	GAGCTCGGTACCAAGCTTAACTAGC
Sanger sequencing	
Zp1 exon4 forward	TCATTGAAACCATTGCCAGCA
Zp1 exon4 reverse	AGGTCTCCTCTGCCATCTG

### 2.6 Protein expression in mammalian cells

293T cells were maintained in high‐glucose DMEM (Gibco) medium supplemented with 10% FBS and grown at 37°C with 5% CO_2_. Transient transfections were performed using PEI reagent (Polyscience). Briefly, for cells at each 10 cm dish, a total of 10 ml fresh complete medium with serum was replenished 30 min prior to transfection. A total of 12 μg tagged Zp plasmids (4 μg each of Flag‐Zp1 or Flag‐Zp1(Mut) in complex with His‐Zp2 and His‐Zp3 plasmids) and 36 μl of PEI were diluted into 500 μl opti‐MEM medium separately. Then, the diluted PEI and plasmids were gently mixed together and incubated for 25 min at RT before addition to the growing cells. Cells were collected 48 hr posttransfection and sonicated in cell lysis buffer supplemented with 1% protease inhibitor cocktail (APE‐BIO).

### 2.7 Immunoblots

Protein extracts were denatured by heating for 10 min at 95°C in SDS‐PAGE sample loading buffer. Proteins were separated by gel electrophoresis, followed by wet transfer to polyvinylidene difluoride (PVDF) membranes (Millipore). After blocking with 5% nonfat milk diluted in phosphate‐buffered saline supplemented with 0.05% Tween 20 for 1 hr. Membranes were probed with primary antibodies using α‐Flag (1:2,000, Proteintech, Cat #66008‐3) and α‐His (1:2,000, Proteintech, Cat #66005‐1). Mouse beta‐actin (1:10,000, Proteintech, Cat # 6600901) was used as internal control. The secondary antibodies were HRP‐conjugated goat anti‐mouse IgG (1:10,000, Abclonal, Cat #AS062). Target proteins were detected using the ECL Western Blotting Detection Kit (Tanon) according to the manufacturer's recommendation.

### 2.8 Co‐immunoprecipitation

Immunoprecipitation assays were performed using 293T cells transfected with Flag‐Zp1 or Flag‐Zp1(Mut), His‐Zp2, and His‐Zp3 plasmids as described above. Total protein lysates from transfected cells were prepared in NP‐40 lysis buffer (20 mM Tris‐HCl [pH 7.5], 200 mM NaCl, 1% NP‐40, and 1% protease inhibitor cocktail [APE‐BIO]), and precleared with protein A Dynabeads (Invitrogen) and protein G Dynabeads (Invitrogen) for 1 hr at 4°C. The precleared extracts (10% for input) were incubated with Flag antibody or mouse IgG overnight at 4°C. Protein A and protein G were added to the antibody extracts mixture followed by incubation for 1 hr at 4°C. Beads were washed with lysis buffer for 4 times prior to elution with SDS sample buffer. Western blotting was conducted as described above.

## CONFLICT OF INTEREST

The authors have declared no conflict of interests.

## AUTHOR CONTRIBUTIONS

J. B. and F. Z. conceived this study; Q.X., Z.W., Z.Z., and Y.C. recruited the patient. X. Z., M. M. W. L., S.K., F.W., and X.M. performed the experiments. J.B. wrote the manuscript with the help of laboratory members. All authors approved the final manuscript.

## Data Availability

The data that support the findings of this study are available on request from the corresponding author.

## References

[mgg31269-bib-0001] Chen, C. , Xu, X. , Kong, L. , Li, P. , Zhou, F. , Zhao, S. , … Zhang, X. (2018). Novel homozygous nonsense mutations in LHCGR lead to empty follicle syndrome and 46, XY disorder of sex development. Human Reproduction, 33(7), 1364–1369. 10.1093/humrep/dey215 29912377

[mgg31269-bib-0002] Chen, T. , Bian, Y. , Liu, X. , Zhao, S. , Wu, K. , Yan, L. , … Chen, Z.‐J. (2017). A recurrent missense mutation in ZP3 causes empty follicle syndrome and female infertility. American Journal of Human Genetics, 101(3), 459–465. 10.1016/j.ajhg.2017.08.001 28886344 PMC5590947

[mgg31269-bib-0003] Coulam, C. B. , Bustillo, M. , & Schulman, J. D. (1986). Empty follicle syndrome. Fertility and Sterility, 46(6), 1153–1155. 10.1016/s0015-0282(16)49898-5 3781029

[mgg31269-bib-0004] Dai, C. , Chen, Y. , Hu, L. , Du, J. , Gong, F. , Dai, J. , … Lin, G. E. (2019). ZP1 mutations are associated with empty follicle syndrome: Evidence for the existence of an intact oocyte and a zona pellucida in follicles up to the early antral stage. A case report. Human Reproduction, 34(11), 2201–2207. 10.1093/humrep/dez174 31734689

[mgg31269-bib-0005] Dai, C. , Hu, L. , Gong, F. , Tan, Y. , Cai, S. , Zhang, S. , … Lin, G. (2019). ZP2 pathogenic variants cause in vitro fertilization failure and female infertility. Genetics in Medicine, 21(2), 431–440. 10.1038/s41436-018-0064-y 29895852

[mgg31269-bib-0006] Liu, C. , Litscher, E. S. , Mortillo, S. , Sakai, Y. , Kinloch, R. A. , Stewart, C. L. , & Wassarman, P. M. (1996). Targeted disruption of the mZP3 gene results in production of eggs lacking a zona pellucida and infertility in female mice. Proceedings of the National Academy of Sciences USA, 93(11), 5431–5436. 10.1073/pnas.93.11.5431 PMC39263 8643592

[mgg31269-bib-0007] Liu, W. , Li, K. , Bai, D. , Yin, J. , Tang, Y. , Chi, F. , … Gao, S. (2017). Dosage effects of ZP2 and ZP3 heterozygous mutations cause human infertility. Human Genetics, 136(8), 975–985. 10.1007/s00439-017-1822-7 28646452

[mgg31269-bib-0008] Mesen, T. B. , Yu, B. , Richter, K. S. , Widra, E. , DeCherney, A. H. , & Segars, J. H. (2011). The prevalence of genuine empty follicle syndrome. Fertility and Sterility, 96(6), 1375–1377. 10.1016/j.fertnstert.2011.09.047 22130102 PMC3576020

[mgg31269-bib-0009] Rankin, T. L. , O'Brien, M. , Lee, E. , Wigglesworth, K. , Eppig, J. , & Dean, J. (2001). Defective zonae pellucidae in Zp2‐null mice disrupt folliculogenesis, fertility and development. Development, 128(7), 1119–1126. 11245577 10.1242/dev.128.7.1119

[mgg31269-bib-0010] Rankin, T. , Talbot, P. , Lee, E. , & Dean, J. (1999). Abnormal zonae pellucidae in mice lacking ZP1 result in early embryonic loss. Development, 126(17), 3847–3855. 10433913 10.1242/dev.126.17.3847

[mgg31269-bib-0011] Revelli, A. , Carosso, A. , Grassi, G. , Gennarelli, G. , Canosa, S. , & Benedetto, C. (2017). Empty follicle syndrome revisited: Definition, incidence, aetiology, early diagnosis and treatment. Reproductive BioMedicine Online, 35(2), 132–138. 10.1016/j.rbmo.2017.04.012 28596003

[mgg31269-bib-0012] Sun, L. , Fang, X. , Chen, Z. , Zhang, H. , Zhang, Z. , Zhou, P. , … Li, N. (2019). Compound heterozygous ZP1 mutations cause empty follicle syndrome in infertile sisters. Human Mutation, 40(11), 2001–2006. 10.1002/humu.23864 31292994

[mgg31269-bib-0013] Wang, K. , Li, M. , & Hakonarson, H. (2010). ANNOVAR: Functional annotation of genetic variants from high‐throughput sequencing data. Nucleic Acids Research, 38(16), e164 10.1093/nar/gkq603 20601685 PMC2938201

[mgg31269-bib-0014] Wassarman, P. M. , & Litscher, E. S. (2018). The mouse egg's zona pellucida. Current Topics in Developmental Biology, 130, 331–356. 10.1016/bs.ctdb.2018.01.003 29853182

[mgg31269-bib-0015] Yuan, P. , He, Z. , Zheng, L. , Wang, W. , Li, Y. U. , Zhao, H. , … Yang, D. (2017). Genetic evidence of 'genuine' empty follicle syndrome: A novel effective mutation in the LHCGR gene and review of the literature. Human Reproduction, 32(4), 944–953. 10.1093/humrep/dex015 28175319

[mgg31269-bib-0016] Zhou, Z. , Ni, C. , Wu, L. , Chen, B. , Xu, Y. , Zhang, Z. , … Wang, L. (2019). Novel mutations in ZP1, ZP2, and ZP3 cause female infertility due to abnormal zona pellucida formation. Human Genetics, 138(4), 327–337. 10.1007/s00439-019-01990-1 30810869

[mgg31269-bib-0017] Zreik, T. G. , Garcia‐Velasco, J. A. , Vergara, T. M. , Arici, A. , Olive, D. , & Jones, E. E. (2000). Empty follicle syndrome: Evidence for recurrence. Human Reproduction, 15(5), 999–1002. 10.1093/humrep/15.5.999 10783341

